# Hemophagocytic Lymphohistiocytosis in Children: Pathogenesis and Treatment

**DOI:** 10.3389/fped.2016.00047

**Published:** 2016-05-13

**Authors:** Eiichi Ishii

**Affiliations:** ^1^Department of Pediatrics, Ehime University Graduate School of Medicine, Toon, Ehime, Japan

**Keywords:** hemophagocytic lymphohistiocytosis, familial, Epstein–Barr virus, genetic defects, cytotoxicity, hematopoietic stem cell transplantation

## Abstract

Hemophagocytic lymphohistiocytosis (HLH) is a rare disorder in children that is characterized by persistent fever, splenomegaly with cytopenia, hypertriglyceridemia, and hypofibrinogenemia. Increased levels of various cytokines and soluble interleukin-2 receptor are biological markers of HLH. HLH can be classified into two major forms: primary and secondary. Familial hemophagocytic lymphohistiocytosis (FHL), a type of primary HLH, is an autosomal recessive disorder that typically occurs in infancy and can be classified into five different subtypes (FHL types 1–5). In Japan, >80% of patients with FHL have either *PRF1* (FHL type 2) or UNC13D (FHL type 3) defects. FHL is considered to be a disorder of T-cell function because the activity of NK cells or cytotoxic T lymphocytes as target cells is usually impaired. Moreover, Epstein–Barr virus-associated HLH (EBV-HLH) is considered a major subtype of secondary HLH. Any genetic background could have an effect on the pathogenesis of secondary HLH because EBV-HLH is considered to be particularly prevalent in Asian countries. For primary HLH, hematopoietic stem cell transplantation is the only accepted curative therapy, although cord blood transplantation with a reduced-conditioning regimen has been used with superior outcomes. For secondary HLH, including EBV-HLH, immunochemotherapy based on the HLH-2004 protocol has been used. In the near future, the entire mechanism of HLH should be clarified to establish less toxic therapies, including cell therapy and gene targeting therapy.

## Introduction

Hemophagocytic lymphohistiocytosis (HLH) in children is characterized by persistent fever, splenomegaly with cytopenia, hypertriglyceridemia, and hypofibrinogenemia. The infiltration of histiocytes with hemophagocytic activity is usually observed in reticuloendothelial systems, including the bone marrow (BM) and central nervous system (CNS) (1). HLH is classified into two major forms: primary and secondary. Primary HLH includes familial hemophagocytic lymphohistiocytosis (FHL) and several primary imunodeficiencies, which exhibit genetic inheritance and usually occur in infancy. Secondary HLH is associated with infections [mainly Epstein–Barr virus (EBV)], autoimmune disorders (mainly juvenile idiopathic arthritis), or malignancies (mainly non-Hodgkin’s lymphoma) (2). The pathogenesis of HLH has been recently clarified to be the impaired activation of T lymphocytes following stimulation by immune responses, resulting in large quantities of inflammatory cytokines that promote macrophage infiltration and cytokine network formation (3).

Several genetic defects in primary HLH, particularly FHL, have been recently identified, which include perforin (*PRF1*), Munc13-4 (*UNC13D*), syntaxin 11 (*STX11*), and Munc18-2 (*STXBP2*) (4–7). In Japan, 80% of patients with FHL have either *PRF1* or *UNC13D* defects, whereas >10% have unknown genetic defects, which should be elucidated in the future (8). Although primary HLH can be controlled by immunochemotherapy, hematopoietic stem cell transplantation (HSCT) is required as a curative therapy. Furthermore, the outcome of EBV-HLH has been superior with immunochemotherapy on the basis of the HLH-94/HLH-2004 protocol. However, some patients who are resistant to conventional therapy also require HSCT.

This review focuses on the recent advances of analysis regarding pathogenesis and treatment of HLH. Identifying all mechanisms underlying HLH could facilitate the development of novel approaches, including cell therapy and gene targeting therapy, in the future.

## Clinical and Laboratory Findings of HLH

The clinical findings of HLH are usually non-specific, as other disorders including severe infections or secondary HLH in infancy display similar clinical findings. The important criteria to diagnose initially as HLH that was proposed by the HLH-2004 study include persistent fever that is resistant to antibiotics and splenomegaly with or without hepatomegaly (Table 1) (9). As other findings, pulmonary infiltrates and pleural effusion were described in a few patients (3). Neurological abnormalities including irritability, depressed consciousness level, hypotonia, and convulsions can be seen especially in FHL. These neurological abnormalities are associated with CNS infiltration of histiocytes or lymphoid cells with hemophagocytic activity (3). Additional symptoms including icterus, ascites, and edema are frequently observed late in the course of HLH.

**Table 1:** **Diagnostic criteria of HLH**.

**1. Only for primary HLH**
Defects of known causable genes
Positive family history
**2. Clinical findings**
Fever
Splenomegaly
Cytopenia: Hb <9.0 g/dl, platelets <100,000/μl, and neutrophils <1,000/μl
Hypertriglyceridemia and/or hypofibrinogenemia
High triglycerides >3.0 mmol/l
Low fibrinogen <150 mg/dl
Hemophagocytosis
**3. Laboratory findings**
Low NK cell activity
Hyperferritinemia (>500 ng/ml)
High sIL-2R (>2,400 U/ml)

Laboratory findings include cytopenia (affecting ≥2 of three lineages in the peripheral blood), hypertriglyceridemia and/or hypofibrinogenemia, liver dysfunction with high LDH, hyperferritinemia, and hemophagocytosis in BM or the reticuloendothelial system (spleen and lymph nodes) (3). Leukocyte count at diagnosis varies among patients, whereas the majority of patients exhibit anemia and/or thrombocytopenia (1). Without treatment, nearly all patients develop severe pancytopenia with progression of the disease, which necessitates frequent transfusions (1, 3). Compared with secondary HLH, hemophagocytosis in BM can detected in only half or two-thirds and repeated BM examinations are necessary for the diagnosis of FHL (3). Pleocytosis with an increased protein level in the cerebrospinal fluid or abnormal radiological findings of the brain that were detected using CT or MRI can be sometimes observed at onset or during the course of the disease, despite the absence of neurological abnormalities. Among them, hypertriglyceridemia and/or hypofibrinogenemia and hyperferritinemia are diagnostic criteria for HLH (Table 1) (9).

Various serum cytokines and chemokines have been used as biological markers of HLH (10). In particular, the levels of tumor necrosis factor (TNF)-α and interleukin (IL)-6, which are secreted by activated monocytes/macrophages, and interferon-γ and soluble CD8 antigens, which are secreted by T lymphocytes, are increased in patients with HLH. The level of soluble IL-2 receptor sCD25 is elevated in patients with HLH, suggesting the activation of T lymphocytes in this disorder. Low or deficient NK cell activity is observed in the majority of patients with FHL, whereas low NK activity occurs in some patients with secondary HLH, which usually returns to normal following treatment (11). Increased soluble IL-2 receptor levels and low or deficient NK activities can serve as diagnostic markers of patients with HLH (Table 1).

## Mechanism of NK/CTL-Mediated Cytotoxicity

**Figure 1:**
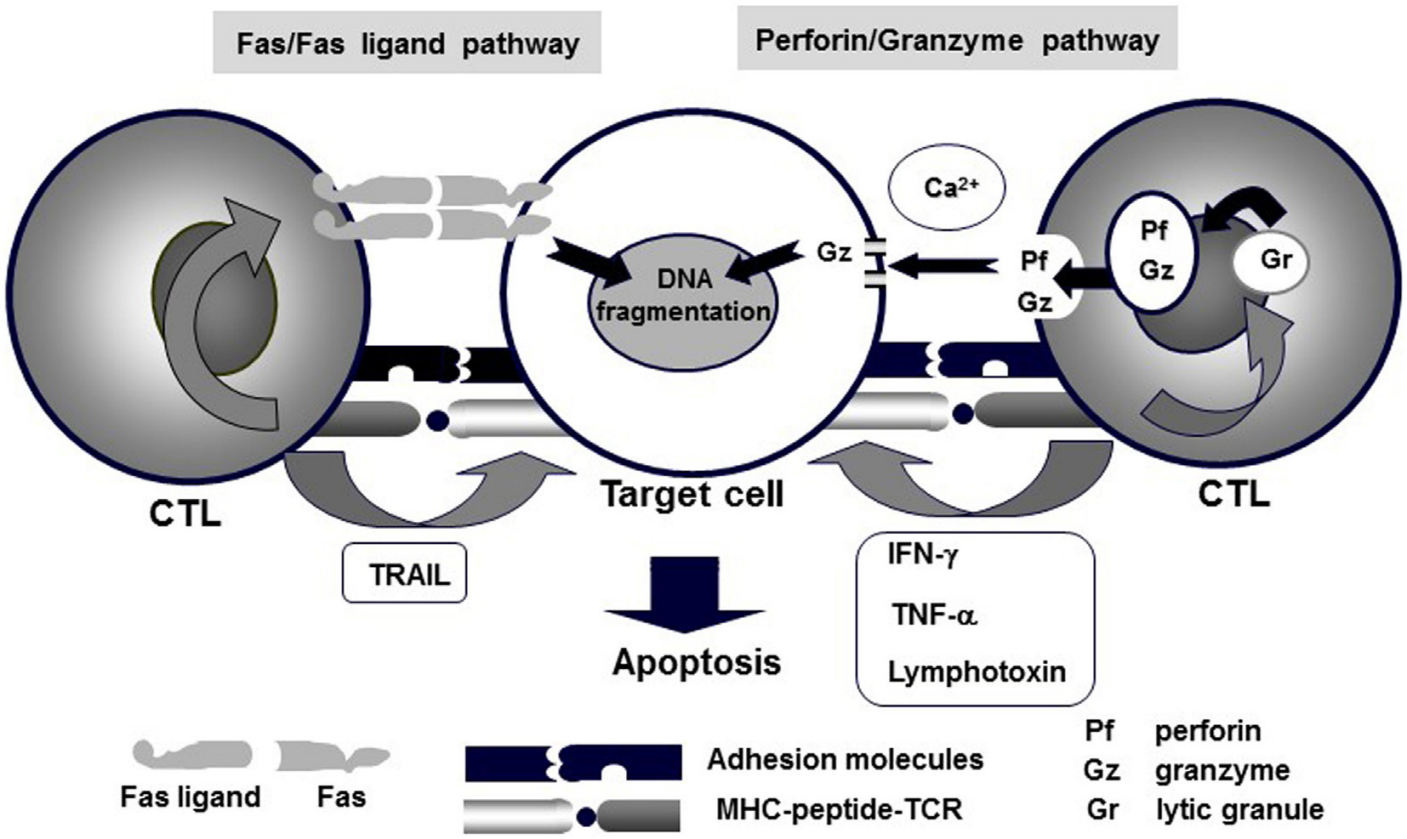
**Mechanisms of CTL-mediated cytotoxicity**. Various pathways, including perforin/granzyme, Fas/Fas ligand, membrane-bound TNF-α, membrane-bound lymphotoxin, and TRAIL, underlie CTL-mediated cytotoxicity. Among these, granule exocytosis that is mediated by the perforin/granzyme and Fas/Fas ligand pathways are believed to be the main mechanisms of CTL-mediated antigen-specific cytotoxicity.

NK/CTL-mediated cytotoxicity plays a central role in the pathogenesis of HLH. Various pathways, including perforin/granzymes, Fas/Fas ligand, membrane-bound TNF-α, membrane-bound lymphotoxin, and TNF-related apoptosis-inducing ligand (TRAIL), have been implicated in NK/CTL-mediated cytotoxicity (12). Among these, two pathways, exocytosis of lytic granules mediated by perforin/granzyme and Fas/Fas ligand interactions, have been identified as the main mechanisms of NK/CTL-mediated antigen-specific cytotoxicity (Figure 1) (13, 14). In particular, the former pathway involves pore formation in the target cell membrane through extracellular Ca^2+^-dependent polymerization of perforin, which is accompanied by the release of serine proteases, granzymes, which are responsible for the downstream activation of caspase-3 and subsequent DNA fragmentation, resulting in target-cell apoptosis (3). The genetic defects in FHL and several primary immunodeficiencies have been identified to code the molecules involved in the perforin/granzyme pathway. In fact, our preliminary studies demonstrated that using CD4^+^ and CD8^+^ CTLs, the perforin/granzyme-dependent cytotoxic pathway is impaired in patients with FHL (15, 16).

**Figure 2:**
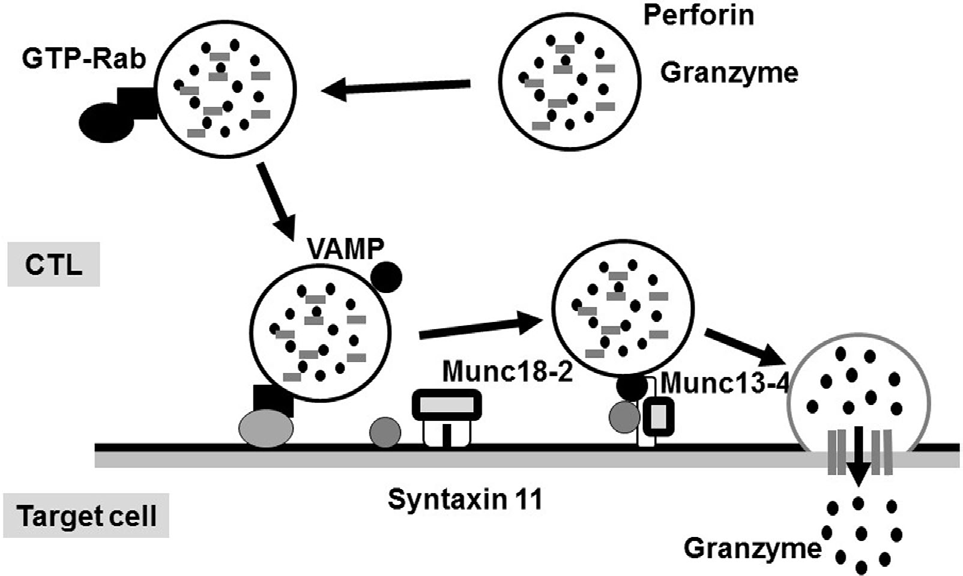
**Secretion of lytic granules in NK/CTL**. After stimulating target cells, CTL organizes the immunological synapse. Rab27a promotes the transporting and docking of lytic granules at the immunological synapse along with Munc13-4. Interactions between Munc18-2 and SNARE (such as syntaxin 11) allow lytic granules to reach the synapse, where granule secretion occurs.

The molecular mechanism underlying the perforin/granzyme pathway is shown in Figure 2 (17). Perforin and granzyme are included in lytic granules of NK/CTLs. The final step of granule transportation is mediated by the immunological synapse between granules and their target membrane through the formation of a complex between a vesicle-SNARE (v-SNARE) protein, such as a VAMP, and a target membrane-SNARE (t-SNARE), such as a syntaxin11 or a member of SNAP23/25/29 (8, 18). The SNARE complex comprises three molecules: VAMP, syntaxin11, and SNAP23/25/29. However, the precise biological functions of the SNARE system remain poorly understood. Recent evidence suggests that members of the SNARE family mediate the fusion of cytotoxic lytic granules with the surface of CTLs. Syntaxin11, SNAP23, and VAMP7 are prime candidates that function as SNAREs in this fusion event (19).

## Genetic Defects and Pathogenesis of FHL

Genetic defects responsible for FHL have been identified by various methods. Linkage analysis has indicated one possible locus of FHL type 1 at 9q21.3–22; however, a causative gene remains to be identified (20). In 1999, a mutation in the perforin gene (*PRF1*) was identified as the cause of FHL type 2 (4). Thereafter, genetic mutations of the Munc13-4 gene (*UNC13D*) as a cause of FHL type 3, syntaxin11 gene (*STX11*) as a cause of FHL type 4, and Munc18-2 gene (*STXBP2*) as a cause of FHL type 5 were identified (5–7). These mutations affect the proteins that are involved in the transportation, membrane fusion, or exocytosis of perforin/granzyme of the lytic granules of NK/CTLs.

The incidence of each subtype varies among countries. Our recent study demonstrated that the actual incidences of FHL types 2 and 3 in Japan were approximately 55 and 32%, respectively, whereas FHL type 5 accounted for only 6%, with no cases of FHL type 4 having yet been reported (8). It is notable that in some cases of FHL, the responsible genetic mutation is unknown. In West Asian countries, mutations of three genes (*PRF1*, *UNC13D*, and *STX11*) were identified in 80% of patients with FHL, while *STXBP2* mutation accounted for only 10% (21). In contrast, in Korea, most patients with FHL had *UNC13D* mutations (22). In North America, the incidence of a *PRF1* mutation is the highest, followed by *UNC13D* and *STXBP2* (23). Thus, the distribution of each FHL subtype varies among different ethnic groups.

In the SNARE system, perforin is critical for granzyme delivery and Munc13-4 is essential for priming of cytotoxic granules that are docked at the immunological synapse, whereas syntaxin11 regulates membrane fusion events (8, 24, 25). *Via* the interaction with syntaxins11, Munc18-2 is required for docking and the fusion of lytic granules with the immunological synapse (26). A recent report indicated that docked granules are primed for fusion by Munc13-4 when Munc18-2 clasps across the zippering 4-helix-assembled trans-SNARE complex (27). These findings suggest that at the immunological synapse, the Munc18-2/syntaxin11/Munc13-4 complex may play a role by regulating granule docking and initiating SNARE formation prior to the priming step (8, 24, 25). These findings support the hypothesis that the cytotoxic activities of NK/CTLs in FHL types 3–5 are impaired to a similar degree.

**Figure 3:**
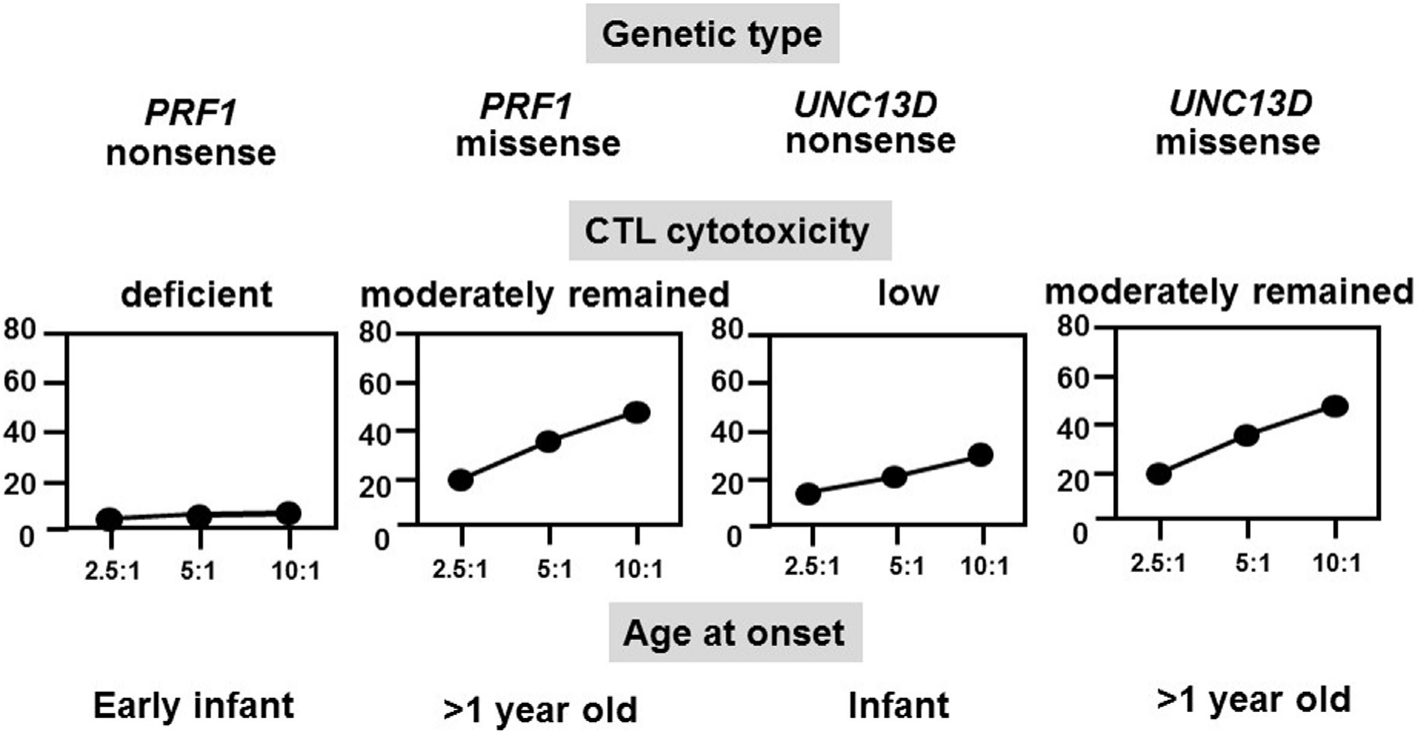
**Correlation among genetic subtype, CTL activity, and age at onset of FHL**. The cytotoxicity of CD8^+^ T-cell lines that were generated from patients with different FHL subtypes was determined. Cytotoxicity was deficient in patients with FHL2 having *PRF1* nonsense mutations and was very low in patients with FHL3 having *UNC13D* nonsense mutations but was moderate in those with missense mutations.

FHL typically occurs within the first year of life in 70–80% of cases, whereas late-onset cases and teenagers with *PRF1* defects have also been described and all had a missense mutation at least in one allele (28). NK cell function was also impaired in the majority of these cases. We analyzed the relationships among genetic defects, CTL activities, and ages at the onset of different FHL subtypes and found that CTL-mediated cytotoxicity was deficient in patients with FHL2 having a *PRF1* nonsense mutation and onset was usually in early infancy, while cytotoxicity was low in patients with FHL3 having an *UNC13D* nonsense mutation and onset also occurred in infancy (Figure 3) (16). However, cases with a genetic missense mutation were associated with moderate CTL-mediated cytotoxicity and the onset was after the age of >1 year, indicating the close association between the type of genetic mutation, degree of CTL cytotoxicity, and age at onset. A few patients with a *PRF1* missense mutation have survived without undergoing HSCT.

## HLH in Primary Immunodeficiencies

HLH develops in various primary immunodeficiencies, including Chediak–Higashi syndrome (CHS), Griscelli syndrome (GS) type 2, Hermansky–Pudlak syndrome (HPS) type 2, and X-linked lymphoproliferative disease (Table 2). Although FHL is most common, these hereditary diseases should be a differential diagnosis of primary HLH. Affected organs are important to distinguish these hereditary diseases. The combination of immunodeficiency and albinism of the skin or hair are observed in CHS, GS type 2, and HPS type 2, but not in FHL.

**Table 2:** **Gene mutations associated with primary HLH**.

	Chromosome	Gene	Protein and/or function
FHL			
FHL 1	9q21.3–22	Unknown	Unknown
FHL 2	10q22	*PRF1*	Perforin: apoptosis and cytotoxicity
FHL 3	17q25.1	*UNC13D*	Munc13-4: exocytosis of granules
FHL 4	6q24	*STX11*	Generation of granules with SNAP23
FHL 5	19p13.3–2	*STXBP2*	Syntaxin-binding protein 2
XLP			
XLP1	Xq25	*SH2D1A*	SAP: activation of lymphocytes
XLP2	Xq25	*XIAP*	XIAP: inhibitor of apoptosis
Griscelli syn II	15q21	*RAB27A*	Exocytosis of granules
Hermansky–Pudlak syn II	5q14.1	*AP3B1*	AP3βchain: traffic from Golgi to granules
Chediak–Higashi syn	1q42.1–2	*LYST*	Transport of lysozome
ADA deficiency	20q13.11	*ADA*	Metabolism of nucleic acids
PHP deficiency	14q13.1	*PNP*	Metabolism of nucleic acids
IL-2Rα chain def.	10p15–14	*IL-2RA*	IL-2R: T cell activation and regulation
Common γ chain def.	Xq13	*IL-2RG*	IL-2R: T cell activation and regulation
Wiskott–Aldrich syn	Xp11.23–22	*WASP*	WASP: cytoskeleton
DiGeorge syn	22q11.2	*DCGR*	Unknown/various
XL-O-EDA-ID	X28	*NEMO*	NEMO protein
XLA	Xq21.3-q22	*BTK*	B cell maturation and proliferation
Hyper-lgD syn	12q24	*MVK*	Mevalonate kinase
Lysinuric pro. intolerance	14q11.2	*SLC7A7*	Transport of amino acid
Multiple sulfatase def.	3p26	*SUMF1*	FGE: transcriptional activation of sulfatase
Cobalamin C disease	1p	*MMACHC*	Metabolism of vitamin B12
Hott–Oram syn	12q24.1	*TBX5*	promotes cardiomyocyte differentiation

Among these immunodeficiencies, only patients with CHS have been reported in the registry system of Japan, while the number of CHS cases has been only one per year. A mutation of the lysosomal trafficking regulator (*LYST*) gene at 1q42–44 is the cause of CHS. *LYST* encodes a cytoplasmic protein that modulates lysosomal exocytosis (29). The LYST protein interacts with some cytoplasmic proteins, such as hepatocyte growth factor-regulated tyrosine kinase substrate (HRS), casein kinase II, calmodulin (CALM), and 14-3-3 proteins, forming a complex that plays an important role in regulating the vesicular transport and signal transduction. HRS inhibits exocytosis by binding SNAP25, a component of the SNARE protein complex that is important in vesicle docking and fusion (30). The LYST protein binds to HRS, forming the HRS–LYST complex, which is unable to bind SNAP25. In CHS, the absence of LYST might prevent juxtaposition of HRS and CALM, thereby potentiating HRS inhibition of SNAP25 and inhibiting membrane docking and fusion (30). The *LYST* defect causes neutrophil digestion and migration and NK/CTL cytotoxicity.

Most patients with CHS develop HLH as an “accelerated phase” during infancy or childhood, whereas some patients exhibit a mild phenotype and survive to adulthood without HLH. A recent study reported that cytotoxic function was significantly reduced in patients with CHS who developed early onset HLH (31). Recently, we analyzed 15 patients with CHS in Japan, of whom 5 (33%) developed life-threatening HLH (32). Five patients, including 3 with HLH, underwent HSCT, and 10 patients survived. *LYST* analysis was performed in nine cases, which identified six different mutations in six patients, while no mutation was found in the remaining three patients. CTL cytotoxicity was particularly impaired in patients who developed HLH. Thus, patients with low CTL cytotoxicity can develop HLH during the course of a disease.

## Epstein–Barr Virus-Associated Hemophagocytic Lymphohistiocytosis

EBV-HLH is a major subtype of secondary HLH that is induced by a primary EBV infection. In a nationwide survey, the most frequent subtype in Japan is EBV-HLH (approximately 40% of all patients with HLH), followed by other infection- or lymphoma-associated HLH (2). EBV-HLH, an EBV infection, will exclusively occur in T and/or NK cells (33). The incidence of EBV-HLH is relatively high in Asian countries, indicating the underlying genetic background in the pathogenesis of EBV-HLH.

The exact mechanism underlying EBV induction of HLH has been elucidated. A previous report suggested that EBV-infected B cells induce the proliferation of cytotoxic T cells followed by the activation of macrophages, resulting in uncontrolled immune activation and induced hypercytokinemia, which is known as a “cytokine storm” (34). Another report suggested that EBV targets CD8^+^ T or NK cells *via* CD21, which is expressed on the surface of these cells, leading to uncontrolled production of cytokines because of widespread lymphohistiocytic activation (35).

Phenotypically, EBV-HLH is a heterogeneous disorder with various symptoms, ranging from mild to severe, and with a variable clinical course, ranging from self-limiting to severe/aggressive or fatal. Therefore, prompt and appropriate treatment should be established according to prognostic factors, which will be addressed later.

T-cell receptor (TCR) gene rearrangement is detectable in half of the patients with EBV-HLH using Southern blotting and/or PCR analyses. We speculate that the presence and change of TCR gene clonality is likely to be associated with the outcome of patients with EBV-HLH. Sandberg et al. (36) recently reported that BIOMED-2 multiplex PCR could replace Southern blot analysis in routine testing of T-cell clonality. We also analyzed TCR gene clonality with BIOMED-2 multiplex PCR in six patients with EBV-HLH (37). All patients showed monoclonal peaks in TCRβ and/or TCRγ at diagnosis, and serial monitoring of one patient disclosed a change in the rearrangement pattern of the TCR genes in response to immunochemotherapy, indicating that BIOMED-2 multiplex PCR, a highly sensitive method for detecting T-cell clonality, is useful to predict the therapeutic response of patients with EBV-HLH (37).

## Neonatal HLH and Herpes Simplex Virus

**Figure 4:**
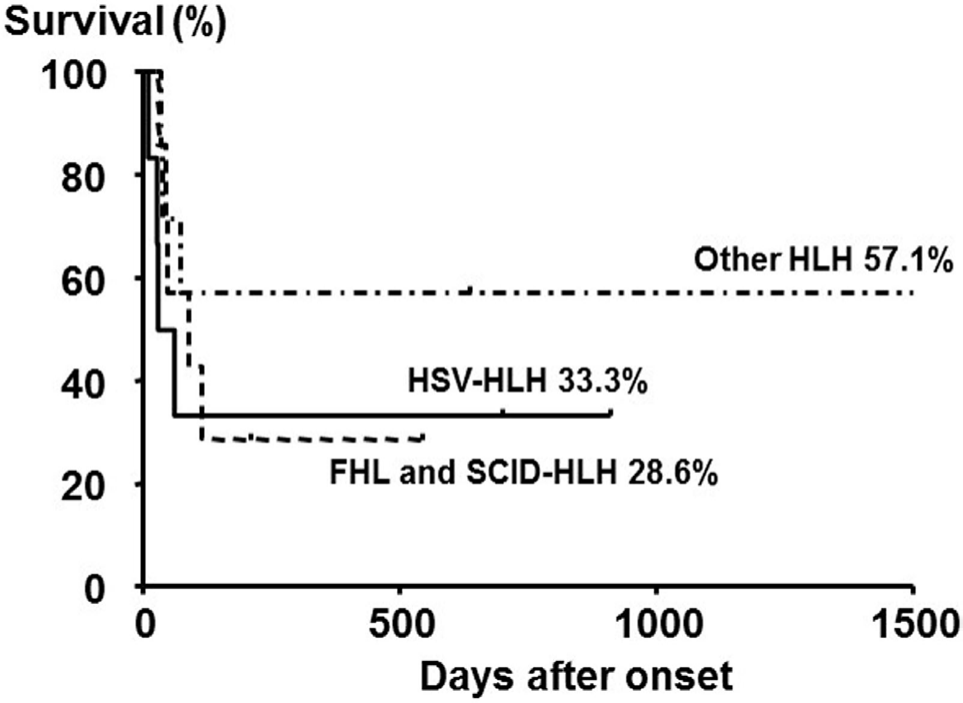
**Outcome of neonatal HLH with different subtypes**. The 5-year overall survival rate of neonates with primary HLH (FHL and SCID-HLH) and HSV-HLH was poor compared with those with other HLH subtypes, although no significant difference was observed among the three groups. Adapted from Suzuki et al. (38). Permission granted by Elsevier.

Neonatal HLH onset within 4 weeks after birth is very rare, and the diagnosis is frequently delayed, made at autopsy, or sometimes not made because of the lack of recognition of the disease. Neonatal HLH can be distinguished from HLH in older children with respect to etiology, manifestations, and laboratory findings. Of 20 neonates (10 males and 10 females) who were diagnosed with HLH within 4 weeks after birth, 6 (30%) were diagnosed with FHL and 6 (30%) were diagnosed with herpes simplex virus-associated HLH (HSV-HLH) (38). The overall survival rate of these 20 patients was 40:28.6% for FHL and SCID-HLH and 33.3% for HSV-HLH, despite the use of acyclovir-containing therapies (Figure 4).

Although HSV or enterovirus are not common pathogens in HLH of older children, fatal or fulminant enterovirus (echovirus and coxsackievirus) and HSV infections sometimes exasperate HLH in neonates (39, 40). Hence, prompt administration of high-dose acyclovir for neonatal HLH before diagnosis by viral studies should be mandatory.

## Treatment of HLH

Today, effective initial therapy of HLH consists of combinations of proapoptotic chemotherapy and immunosuppressive drugs targeting the hyperactivated T cells and histiocytes (41). Most of the HLH patients have severe systemic symptoms at diagnosis, and timely appropriate treatment for HLH is needed before genetic testing to distinguish primary from secondary HLH. Early use of γ-globulin and/or corticosteroid is sometimes useful to control the activity of HLH, with transient effect (1). However, an aggressive therapeutic approach is warranted in most cases, including immunochemotherapy and HSCT.

The HLH-94 and HLH-2004 studies were both mainly organized for treating primary HLH by HSCT. The first trial HLH-94 study included dexamethasone, cyclosporine A, and etoposide for 8-week-induction therapy, followed by HSCT in patients with familial, persistent, or recurrent disease (42). The results were reported elsewhere (43). The 5-year survival rate was 54 ± 6% in all patients, and 66 ± 8% in those undergoing HSCT. Notably, 72 patients (29%) died during the first 2 months before HSCT, mostly because of disease reactivation, particularly progression of CNS disease (43). The second trial HLH-2004 study also included dexamethasone, cyclosporine A, and etoposide; compared with HLH-94 study, cyclosporine A was used at the onset of disease in HLH-2004 study, and intrathecal therapy with methotrexate and corticosteroids was added in selected patients (9). Although the treatment results have not been still published, the number of patients who died during the first 2 months before HSCT has been reduced compared that of HLH-94 study.

**Figure 5:**
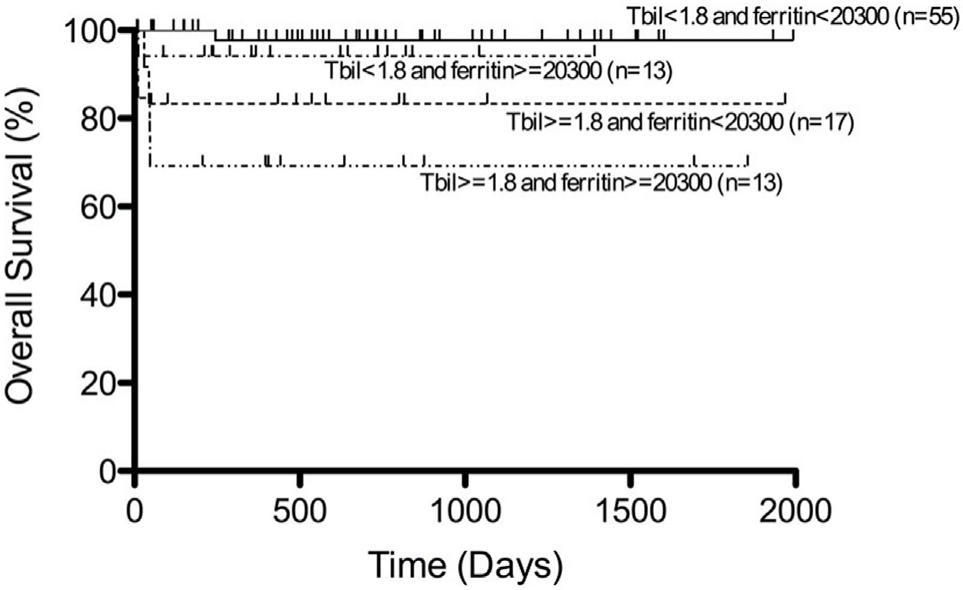
**Survival of patients with EBV-HLH**. Outcome of patients with EBV-HLH having both hyperbilirubinemia and hyperferritinemia at diagnosis was significantly poorer than for those having low bilirubin and ferritin levels. Adapted from Kogawa et al. (44). Permission granted by John Wiley and Sons.

Most of the patients with secondary HLH including EBV-HLH have been registered and treated according to the HLH-94 or HLH-2004 studies. In our recent analysis, more than half of the patients with EBV-HLH were treated with immunochemotherapy based on the HLH-94/HLH-2004 protocol, while 30% were treated with a corticosteroid-based therapy and 10% with only supportive therapy, resulting in complete remission in 90% of patients following initial therapy. In these patients, recurrence was observed in only 8.3% and remission was achieved again with additional therapy (44). Among several prognostic factors, patients with both hyperbilirubinemia (>1.8 mg/dl) and hyperferritinemia (>20,300 ng/ml) at the time of diagnosis had significantly poorer outcomes than those with low serum bilirubin and ferritin levels (Figure 5) (44). The presence and change of T-cell clonality are also good markers to predict treatment response, as described above. EBV-HLH is a heterogeneous disorder with various symptoms and clinical courses. Therefore, we recommend initial treatment with high-dose γ-globulin and/or corticosteroid therapy for all patients with EBV-HLH, followed by immunochemotherapy for those resistant to initial therapy or with several risk factors. HSCT is also optional for relapsed cases. Recent experience suggests that reduced-intensity conditioning (RIC) regimes can improve the outcome of patients with HLH undergoing allogeneic HSCT, despite a significant incidence of mixed chimerism (45, 46).

In Japan, 57 patients (43 with FHL and 14 with EBV-HLH) underwent HSCT between 1995 and 2005 (47). Unrelated donor cord blood transplantation (UCBT) was employed in half of these cases and RIC regimen was used in 26%. The overall survival rate was 65.0% for FHL and 85.7% for EBV-HLH. Prognosis was better for EBV-HLH than for FHL after HSCT because of the high incidence of early treatment-related deaths in FHL. We compared the profiles of patients with FHL who survived with those who died and found no significant differences (Table 3) (47). The survival rate of patients undergoing UCBT with RIC was almost comparable with that of those undergoing other transplantation or myeloablative conditioning. Therefore, UCBT with RIC might be acceptable as optimal HSCT for FHL. Considering the high frequency of rejection in UCBT, the use of high-dose melphalan (≥120 mg/m^2^) and additional modifications, including antithymocyte globulin (ATG) or low-dose total-body irradiation, may be required to attain a stable chimera (48).

**Table 3:** **Profiles of survived and died FHL patients**.

	Survived (*n* = 29)	Died (*n* = 14)	*p*-value
Males/females	13:16	10:4	ns
Age at onset (mean, range)	0.6 (0.1–12 years)	0.3 (0.1–0.8 years)	ns
FHL2:FHL3	7:5	5:3	ns
Manifestation at diagnosis (%)			
Fever	96 (27/28)	93 (13/14)	ns
Hepatosplenomegaly	82 (23/25)	93 (13/14)	ns
Lymphadenopathy	29 (8/28)	7 (1/14)	ns
Skin eruption	21 (6/28)	0 (0/14)	ns
CNS abnormality	21 (6/28)	21 (3/14)	ns
Respiratory failure	14 (4/28)	14 (2/14)	ns
DIC	25 (7/28)	50 (7/14)	ns
CNS signs at HSCT (%)	68 (19/28)	64 (9/14)	ns
CSF pleocytosis	44 (7/16)	0 (0/6)	ns
MRI abnormality	52 (13/25)	50 (7/14)	ns
Convulsion	37 (10/27)	80 (7/14)	ns
Disturbed consciousness	26 (7/27)	21 (3/14)	ns
HSCT			
Age at HSCT (mean, range)	1.7 (0.4–15 years)	1.1 (0.7–4.8 years)	ns
UGBT:others	11:6	6:7	ns
MAC:RIC	20:7	9:5	ns

*CNS, central nerves system; HSCT, hematopoietic stem cell transplantation; CSF, cerebrospinal fluid; UCBT, unrelated cord blood transplantation; MAC, myeloabrative conditioning; RIC, reduced-intensity conditioning; ns, not significant*.

The effectiveness of the addition of rituximab to the initial treatment in the HLH-2004 study was recently reported in the US (49). Because EBV initially infects B cells, rituximab is considered effective as an early treatment for EBV-HLH. Moreover, a recent report suggested that B cells may also be targeted in EBV-associated disease; thus, directed therapy with rituximab may reduce the morbidity and mortality by reducing the population of circulating B cell and EBV load. ATG-based immunotherapy of FHL has also been reported to be successful with an acceptable toxicity when used as a first treatment of familial HLH (50). The use of ATG to target EBV-infected T cells may merit investigative trial in the near future.

The other salvage therapies have not established for refractory HLH. Recently, based on the critical role of T cells in HLH pathogenesis, Alemtuzumab, a monoclonal antibody to CD52, had significant response against refractory HLH (23, 51). However, CMV reactivation and adenoviremia were frequent complications of this therapy. The effect of ATG is still controversial for refractory HLH and accumulation of patients who will be treated with ATG in the future.

Supportive care is important during the course of treatment for HLH which includes the prophylaxis of *Pneumocystis jirovecii*, fungal, or oppotunistic infection, and empiric broad-spectrum antibiotics or antifungal therapy should be initiated (23). To increase neutrophil counts, granulocyte-colony stimulating factor (G-CSF) can be also used for myelosuppression.

## Future Directions

In HLH, appropriate diagnosis and prompt treatment according to each subtype are necessary. To date, there is limited evidence of any major advantage of prompt HSCT with regard to the survival of patients with HLH. The HLH-2004 study recommends HSCT only for familial disease or severe and persistent non-familial disease. Early identification of genetic defects and impaired NK/CTL cytotoxicity may also support the use of the HLH-2004 protocol for primary HLH. After identifying genetic abnormalities in FHL, the association among phenotypes, genotypes, and cytotoxic functions of CTLs will be helpful in establishing appropriate timings of HSCT. In patients in whom no genetic defect can be identified, samples should be used for identifying unknown genes that are responsible for other FHL subtypes. The clinical outcomes of HLH are dependent on how rapidly a diagnosis is established. In the near future, establishing appropriate individualized therapies, including cell therapy and gene targeting therapy, is expected.

## Author Contributions

EI takes primary responsibility for the entirety of this review article.

## Conflict of Interest Statement

The author declares that there are no commercial or financial relationships that could be construed as a potential conflict of interest.
